# Familial hematuria

**DOI:** 10.1007/s00467-007-0622-z

**Published:** 2009-10-01

**Authors:** Clifford E. Kashtan

**Affiliations:** Department of Pediatrics, Division of Pediatric Nephrology, University of Minnesota Medical School, University of Minnesota Children’s Hospital, Fairview, 420 Delaware Street SE, MMC 491, Minneapolis, MN 55455 USA

**Keywords:** Hematuria, Alport syndrome, Thin basement membrane nephropathy, Type IV collagen

## Abstract

Hematuria is a common presenting complaint in pediatric nephrology clinics and often has a familial basis. This teaching article provides an overview of causes, diagnosis, and management of the major forms of familial hematuria, Alport syndrome, and thin basement membrane nephropathy.

## Introduction

The typical pediatric nephrologist will evaluate hundreds, if not thousands, of children and adolescents with hematuria during the course of her or his career. The major forms of familial hematuria, Alport syndrome and thin basement membrane nephropathy (TBMN), together account for approximately 30–50% of children with isolated glomerular hematuria referred to pediatric nephrology clinics for consultation [1–4]. The objectives of this teaching article are to (1) assist pediatric nephrologists in recognizing patients who may have familial forms of hematuria, (2) suggest diagnostic approaches that can be used to confirm familial hematuria and make specific diagnoses, and (3) discuss the implications of a diagnosis of familial hematuria for prognosis, reproductive counseling, and treatment.

## What is familial hematuria?

For this article, familial hematuria is defined as a group of genetic disorders of glomerular capillaries that are characterized clinically by the onset of persistent hematuria during childhood. Hematuria may be the presenting feature of other heritable diseases, including polycystic kidney disease, hypercalciuria, and other forms of hereditary urolithiasis. This article will focuses on the major forms of familial hematuria: Alport syndrome and TBMN. All patients with Alport syndrome and about 50% of those with TBMN have mutations in type IV collagen, the predominant collagenous constituent of glomerular capillary walls. Minor forms of familial hematuria include Epstein and Fechtner syndromes, which arise from mutations in nonmuscle myosin heavy chain IIA [5]. In these autosomal dominant disorders, hereditary nephritis with ultrastructural features that may be reminiscent of Alport syndrome is associated with sensorineural deafness and macrothrombocytopenia.

## Genetics of familial hematurias

A brief review of the genetics of the familial hematurias may be helpful at this point (Table 1). About 80% of those with Alport syndrome have the X-linked form of the disease (XLAS), which is caused by mutations in *COL4A5*, the gene encoding the α5 chain of type IV collagen [α5(IV)]. Autosomal recessive Alport syndrome (ARAS) accounts for about 15% of affected individuals and arises from mutations in both alleles of *COL4A3* or *COL4A4*, which respectively encode the α3(IV) and α4(IV) chains. The remainder of those with Alport syndrome, about 5%, has autosomal dominant Alport syndrome (ADAS) due to heterozygous mutations in *COL4A3* or *COL4A4*. However, the majority of those with heterozygous mutations of *COL4A3* or *COL4A4* have a form of familial hematuria that is usually nonprogressive: TBMN.

**Table 1:** Familial hematurias

	Locus	Protein
Alport syndrome		
X-linked	COL4A5	α5(IV)
Autosomal recessive	COL4A3	α3(IV)
	COL4A4	α4(IV)
Autosomal dominant	COL4A3	α3(IV)
	COL4A4	α4(IV)
Thin basement membrane nephropathy		
Autosomal dominant	COL4A3	α3(IV)
	COL4A4	α4(IV)
Epstein/Fechtner syndromes		
Autosomal dominant	MYH9	Nonmuscle myosin heavy chain IIA

## Clinical features of familial hematurias

Persistent microscopic hematuria is the hallmark of Alport syndrome, occurring in 100% of boys and 95% of girls with XLAS [6] and all boys and girls with ARAS. Typically, red cells number in the tens to dozens, particularly in boys. Red blood cell casts may or may not be observed. Episodic gross hematuria is not unusual. Some affected boys and girls exhibit nearly continuous gross hematuria. The onset of hematuria in affected boys occurs during infancy. On occasion, an affected boy presents with bloodstains in his diaper. About 10–15% of children with XLAS have de novo mutations. Consequently, the great majority of affected children with XLAS have a parent with hematuria. However, normal urinalyses in the parents of a child with hematuria do not exclude a diagnosis of XLAS. XLAS is unlikely if hematuria is found in the father of a boy with hematuria but not his mother.

Each parent of a child with ARAS is a heterozygous carrier of a mutation in *COL4A3* or *COL4A4*. About 50% of these carriers exhibit hematuria. Therefore, hematuria may be found in both, one, or neither parent of a child with ARAS.

As TBMN is, with rare exceptions, a nonprogressive disorder, a family history of dialysis or kidney transplantation in a child with hematuria suggests a diagnosis of Alport syndrome. When assessing the significance of a family history of renal failure, the typical natural history of Alport syndrome and the evolving epidemiology of chronic kidney disease should be considered. For example, renal failure in a young male relative has different implications than renal failure in an older individual with type 2 diabetes. A family history of deafness should also raise suspicion for Alport syndrome, but it should be kept in mind that any individual whose deafness is due to Alport syndrome will also exhibit renal involvement, and the degree of hearing loss tends to parallel the severity of the kidney disease. So, for example, deafness in an otherwise healthy 65-year-old male relative is probably secondary to a process other than Alport syndrome.

Deafness ultimately develops in 80% of males with XLAS [7] and at least that percentage of boys and girls with ARAS. However, detectable hearing loss is often absent when children with Alport syndrome are first evaluated, because the onset of measurable deficits in hearing typically occurs in late childhood. Hearing loss associated with Alport syndrome has several consistent characteristics: it is not present at birth; it is bilateral, with symmetrical onset and severity; it initially involves high-frequency wavelengths; and gradually extends into conversational speech over time. Consideration of these features of the Alport hearing defect can help prevent the clinician from being led down the wrong diagnostic path when presented with a child who has renal disease and deafness.

About 40% of males with XLAS exhibit characteristic ocular anomalies [7]. A distinctive maculopathy consisting of whitish or yellowish flecks distributed around the macula occurs in approximately 20% of XLAS males [7]. These perimacular flecks increase in number with age [8] and are most likely to be observed by an experienced ophthalmologist on dilated exam. Anterior lenticonus, the protrusion of the central area of the lens into the anterior chamber, is pathognomonic of Alport syndrome and occurs in about 20% of affected males with XLAS [7]. Anterior lenticonus is typically discovered during adolescence or young adulthood. A particularly bothersome problem is recurrent corneal erosion, resulting in acute, severe ocular pain [9]. The maculopathy is found in about 15% of females with XLAS; anterior lenticonus is rarely observed [6]. Patients with ARAS can display the same ocular anomalies as those found in XLAS patients [10].

Because TBMN is, according to our current understanding, a renal-limited disorder, the presence of extrarenal abnormalities such as deafness or ocular lesions in someone with hematuria makes a diagnosis of TBMN unlikely. The absence of such findings does not, however, rule out Alport syndrome, especially in the young patient.

In a small number of families, XLAS is associated with smooth-muscle tumors, or leiomyomata, of the esophagus, tracheobronchial tree and, in females, the external genitalia [11]. The Alport syndrome diffuse leiomyomatosis complex is a contiguous gene syndrome resulting from deletions involving the adjacent *COL4A5* and *COL4A6* genes on the X chromosome [12].

## Renal histology in familial hematurias

Alport syndrome and TBMN may be indistinguishable from each other by routine light and immunofluorescence microscopy, and by electron microscopy, in children. Whereas males with Alport syndrome will eventually exhibit light microscopy abnormalities, females may have normal-appearing kidneys by light microscopy, as with individuals with TBMN. In children with familial hematuria, the presence of mesangial proliferation or focal segmental glomerulosclerosis on light microscopy suggests a diagnosis of Alport syndrome.

Since the early 1970s, electron microscopy has been an important technique for diagnosing both TBMN and Alport syndrome and for differentiating the two conditions in patients with hematuria. Individuals with TBMN display diffuse glomerular basement membrane (GBM) thinning associated with attenuation of the lamina densa. The endothelial and epithelial aspects of GBM are smooth and regular, and podocyte foot processes are intact. GBM attenuation is also the earliest glomerular abnormality in patients with Alport syndrome. As a result, TBMN and Alport syndrome may not be distinguishable by electron microscopy in young children.

The pathognomonic ultrastructural lesion of Alport syndrome consists of (1) thickening of the GBM; (2) splitting of the lamina densa into multiple strands that enclose electron-lucent areas, which may contain electron-dense particles; (3) scalloping of the epithelial aspect of the GBM; and (4) partial to complete disappearance of podocyte foot processes in regions of GBM thickening [13]. In males with XLAS, these changes typically first appear during childhood, and the extent of GBM displaying these alterations increases progressively with age [14]. In females, the extent of GBM thickening ranges from focal to diffuse, and the impact of aging on GBM thickening is unpredictable.

Immunostaining of kidney and skin biopsy specimens using monospecific antibodies against type IV collagen chains is a valuable diagnostic modality in patients with hematuria. The utility of this approach derives from the effects of mutations in *COL4A3*, *COL4A4*, and *COL4A5* genes on expression of α3(IV), α4(IV), and α5(IV) chains in basement membranes. The α3(IV) and α4(IV) chains exist in basement membranes in a single construct: the α3α4α5(IV) heterotrimer. In the normal kidney, the α3α4α5(IV) trimer is present in GBM, Bowman’s capsules, and the basement membranes of distal tubules. Mutations in both alleles of *COL4A3* or *COL4A4* in ARAS patients frequently result in complete absence of α3α4α5 trimers from renal as well as ocular and cochlear basement membranes [15–17]. The α5(IV) chain participates in two trimeric species: the aforementioned α3α4α5(IV) trimer and the α5α5α6 (α5_2_α6) trimer. In the kidney, α5_2_α6 trimers are normally found in Bowman’s capsules, distal and collecting tubule basement membranes, and epidermal basement membranes. Hemizygous *COL4A5* mutations in males with XLAS usually lead to elimination of both α3α4α5 and α5_2_α6 trimers from all basement membranes. *COL4A3* and *COL4A4* mutations do not affect the expression of α5_2_α6 trimers, however.

How can this information be applied to diagnosis of hematuria? In many cases, consideration of type IV collagen immunostaining results can lead to definitive diagnoses:

1. XLAS: In about 80% of males with XLAS, renal basement membranes exhibit complete absence or, at times, markedly diminished immunostaining for α3, α4, and α5(IV) chains, and epidermal basement membranes are negative for α5(IV) chains [18, 19]. About 60–70% of heterozygous females display mosaic staining of renal basement membranes for α3, α4, and α5(IV) chains and of epidermal basement membranes for α5(IV) chains [20, 21]. Clearly, normal immunostaining results cannot exclude a diagnosis of XLAS.
2. ARAS: In many patients with ARAS, GBM is completely negative for α3, α4, and α5(IV) chains [22]. However, Bowman’s capsules and distal and collecting tubules remain positive for α5(IV), because in those basement membranes, α5(IV) is present in the form of α5_2_α6 trimers. For the same reason, epidermal basement membranes also remain positive for α5(IV).
3. TBMN: Immunostaining for type IV collagen is normal in kidneys and skin of subjects with TBMN. As noted above, normal type IV collagen immunostaining does not exclude Alport syndrome. However, normal type IV collagen immunostaining supports a diagnosis of TBMN in those with hematuria, normal urine protein excretion, negative family history of renal failure, and diffuse GBM thinning. Patients with autosomal dominant Alport syndrome also exhibit normal immunostaining of skin and kidney for type IV collagen chains.

## Molecular diagnosis of familial hematurias

The *COL4A3*, *COL4A4*, and *COL4A5* genes have been completely sequenced. Although the genes are large, mutation detection rates in patients with XLAS and ARAS are high [23–25]. Access to laboratories offering type IV collagen gene analysis varies from country to country. Molecular approaches may eventually supersede histological methods for diagnosis of familial hematurias but, for now, renal biopsy and skin biopsy are the tools most clinicians rely upon for diagnosing these conditions. Current information regarding molecular testing for Alport Syndrome can be obtained at www.genereviews.org.

## Renal transplantation in Alport syndrome

In general, patients with Alport syndrome have good transplant outcomes, with graft survival rates comparable with those of patients with congenital urinary tract anomalies [26]. Transplant preparation and posttransplant care are the same for Alport patients, with two important exceptions: First, planning for transplantation requires special care to insure that affected family members are not inadvertently allowed to serve as kidney donors. Donor selection in Alport families is based on a thorough understanding of disease genetics and is discussed in detail in a recent review [27]. The dilemma most frequently encountered during transplant planning for Alport patients involves the decision about whether to allow a heterozygous female with XLAS to donate a kidney to an affected male relative. Because females with XLAS are at significant risk for developing end-stage renal disease (ESRD) late in life, kidney donation should be discouraged, especially in those younger than 45 years of age [6, 28].

Following transplantation, care providers must maintain a high degree of suspicion for posttransplant anti-GBM nephritis as a cause of allograft dysfunction, especially in the first year [27]. Posttransplant anti-GBM nephritis affects about 3–5% of transplanted Alport males, and usually results in rapid allograft destruction despite aggressive therapy [27]. Surveillance for posttransplant anti-GBM nephritis combines routine measures, such as frequent measurement of serum creatinine and a low threshold for performing an allograft biopsy, with serial monitoring for circulating anti-GBM antibodies by enzyme-linked immunosorbent assay (ELISA). It should be noted that a negative anti-GBM ELISA result does not rule out posttransplant anti-GBM nephritis [29], because the commercial ELISA assays are relatively insensitive to anti-α5(IV) antibodies, which make up the bulk of anti-GBM antibodies in patients with posttransplant anti-GBM nephritis [30].

## Management of familial hematurias

Results of studies in transgenic mice and in dogs with Alport syndrome suggest that interference with the generation or action of angiotensin II may slow progression to ESRD [31–33]. However, it must be cautioned that the murine studies may be confounded by strain effects [34], and there have been no controlled studies of angiotensin blockade in human Alport subjects [35]. Uncontrolled studies suggested that cyclosporine might have therapeutic benefit in Alport syndrome [36], but enthusiasm has been dampened by a subsequent study indicating that cyclosporine treatment might be associated with accelerated development of interstitial fibrosis [37].

In the absence of data, administration of angiotensin antagonists, such as angiotensin-converting enzyme (ACE) inhibitors and angiotensin receptor blockers, alone or in combination, to patients who have developed overt proteinuria is a reasonable approach with relatively little capacity for harm. A recent uncontrolled study reported that addition of an aldosterone inhibitor may augment the effect of angiotensin blockade on proteinuria in children with Alport syndrome [38].

Although patients given a diagnosis of TBMN do not need treatment, they should not be lost to follow-up. A reasonable approach would consist of annual urinalysis and blood pressure measurement. As long as blood pressure and urine protein excretion remain in the normal range, no further evaluation is necessary.

## Diagnostic approach

Alport syndrome and TBMN should be in the initial differential diagnosis of all children and adolescents who present with hematuria. Diagnostic criteria for Alport syndrome and TBMN are presented in Table 2.

**Table 2:** Diagnostic criteria for familial hematurias

Alport syndrome
The presence of two of the following diagnostic criteria establishes the diagnosis of Alport syndrome:
Family history of hematuria progressing in males to ESRD
• About 10–15% of males with XLAS have de novo mutations, so family history may be negative for renal disease. Family history may also be negative in patients with ARAS, although one or both parents may have hematuria
Characteristic thickening of the glomerular basement membrane and splitting of the lamina densa, detected by electron microscopy of kidney biopsy specimens
• Children with Alport syndrome may exhibit only diffuse GBM attenuation, making differentiation from TBMN a challenge
Progressive, high-frequency sensorineural deafness
The hearing deficit is frequently detectable by audiometry in later childhood (5–10 years of age) in boys with XLAS and both boys and girls with ARAS
Anterior lenticonus or perimacular retinal flecks
• These changes are pathognomonic of Alport syndrome. Anterior lenticonus is usually not detectable until later adolescence
Characteristic abnormalities of renal basement membrane expression of type IV collagen α3, α4, and α5 chains (i.e. the α3α4α5 network) by immunostaining of renal biopsy specimens
• *It is important to note that normal renal basement membrane expression of type IV collagen α3, α4, and α5 chains cannot by itself exclude a diagnosis of Alport syndrome*
• XLAS: about 80% of XLAS males exhibit complete absence of α3, α4, and α5 chains in renal basement membranes; 60–70% of XLAS females exhibit mosaic staining patterns for these proteins
• ARAS: the characteristic pattern is complete absence of α3, α4, and α5 chains in GBM, absence of α3 and α4 chains in Bowman’s capsules and distal tubule basement membranes, but persistence of α5 chains in Bowman’s capsules and distal tubule basement membranes (as a component of α5α5α6 networks)
• ADAS: results of immunostaining for α3, α4, and α5 chains are normal
Characteristic abnormalities of epidermal basement membrane expression of the α5 chain of type IV collagen by immunostaining of skin biopsy specimens
• The normal epidermal basement membrane expresses α5α5α6 networks. The α3α4α5 network is not expressed in epidermal basement membranes of normal subjects
• *It is important to note that normal epidermal basement membrane expression of the α5 chains cannot by itself exclude a diagnosis of Alport syndrome*
• XLAS: about 80% of XLAS males exhibit complete absence of α5 chains in epidermal basement membrane; 60–70% of XLAS females exhibit mosaic staining patterns for this protein
• ARAS and ADAS: results of immunostaining for the α5 chain in epidermal basement membrane are normal
Mutations in *COL4A3, COL4A4*, or *COL4A5* genes
• XLAS: the rate of detection of mutations in *COL4A5* in males with XLAS is 80–90% by direct sequencing
• ARAS: direct sequencing identifies ∼90% of mutations in *COL4A3* or *COL4A4* in ARAS patients with consanguineous parents
Thin basement membrane nephropathy
A diagnosis of TBMN can be made on the basis of clinical and pedigree data; renal biopsy may be unnecessary. Criteria for a clinical diagnosis of TBMN include *all* of the following criteria:
Isolated hematuria; kidney function, urine protein excretion, and blood pressure are normal
Positive family history of hematuria, consistent with autosomal dominant transmission
Negative family history of kidney failure
Renal biopsy may be used to confirm a suspected diagnosis of TBMN. Histological diagnosis is based upon the finding of diffuse attenuation of GBM, as determined by electron microscopy, and the absence of other abnormalities by light microscopy (glomerular cellular proliferation or glomerulosclerosis), immunofluorescence microscopy (glomerular deposition of immunoglobulin or complement), or electron microscopy (lamina densa splitting or fusion of glomerular visceral epithelial cell foot processes). Diffuse GBM attenuation is present when mean GBM width is greater than 2 SD below age- and gender-specific mean values.
Immunostaining of renal biopsy specimens for type IV collagen chains can provide useful adjunctive data. In a patient with diffuse GBM attenuation and who satisfies clinical criteria for TBMN, normal immunostaining for type IV collagen α3, α4, and α5 chains (i.e. the α3α4α5 network) supports a diagnosis of TBMN.

In general, any child whose hematuria does not clearly localize to the lower urinary tract may have familial hematuria. A careful family history aimed at identifying relatives with known hematuria, kidney failure, or deafness is an essential feature of the evaluation of children with hematuria. However, it must be remembered that a negative family history does not exclude Alport syndrome or TBMN. It is also useful to screen all members of the child’s immediate family (parents and siblings) for hematuria, as people with microscopic hematuria may not know they have it.

Does every child with glomerular hematuria need a kidney biopsy, or skin biopsy? The answer is “probably not”. A child with dominantly inherited, isolated glomerular hematuria and a negative family history of kidney failure may be given a tentative diagnosis of TBMN and followed expectantly. In a family with a well-established diagnosis of Alport syndrome, children with hematuria may be presumed to be affected, although it is the author’s practice to obtain a renal ultrasound in every affected child to exclude a structural abnormality of the urinary tract that could affect prognosis.

**Fig. 1:**
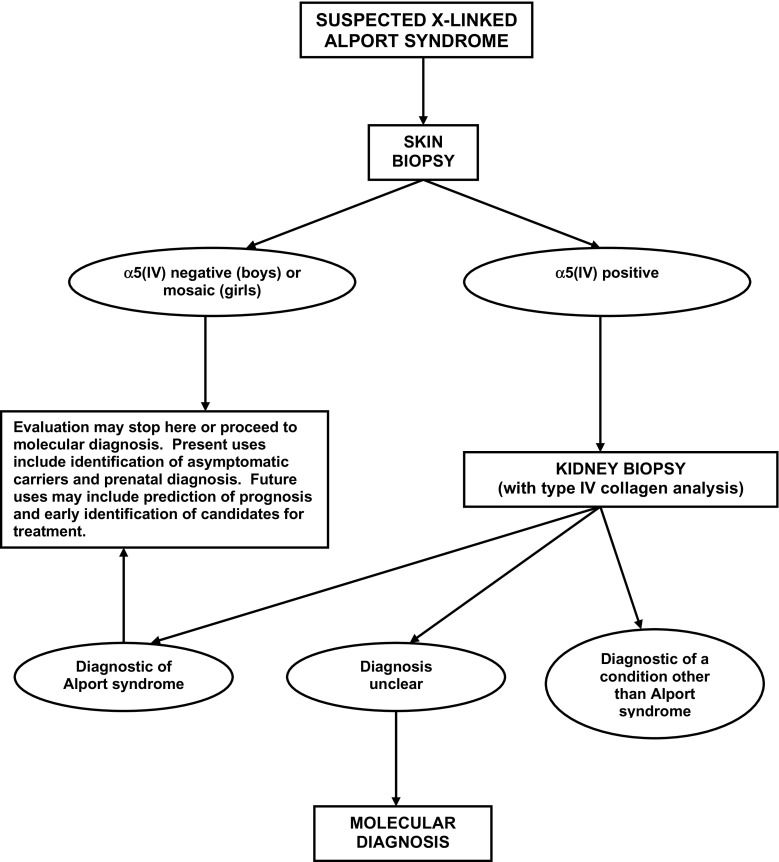
Algorithm incorporating skin biopsy, kidney biopsy, and selective use of molecular diagnostic methods

Skin biopsy with immunostaining for the α5(IV) chain is particularly useful when suspicion of XLAS is high. Examples include a child with hematuria and a positive family history of hematuria and kidney failure, or a child with hematuria and sensorineural deafness. An algorithm incorporating skin biopsy, kidney biopsy and selective use of molecular diagnostic methods is presented in Fig. 1. When XLAS is just one of several possible diagnoses, for example in a child with hematuria and negative family history, a kidney biopsy is more likely to yield a specific diagnosis.

Molecular diagnosis is likely to play a burgeoning role in the diagnostic evaluation of hematuria, although access and expense are still important limitations to this approach. The importance of very early diagnosis may increase if it can be shown that very early pharmacologic intervention can modify the natural history of Alport syndrome. Molecular diagnosis would be ideal for this purpose, although skin and kidney biopsy are also applicable.

## Summary

An early and accurate diagnosis in children with familial hematuria provides important information for genetic counseling, allays unnecessary anxiety in those with TBMN, and identifies those who may benefit from intervention. Careful assessment of clinical findings, pedigree data, and results of tissue studies allow accurate diagnosis in the majority of patients. Wider availability of molecular diagnosis would increase diagnostic precision.

**Questions** (Answers appear following the reference list)

1. Alport syndrome is a disorder of
  - type I collagen
  - type II collagen
  - type IV collagen
  - type V collagen
  - type VI collagen
2. The percentage of Alport patients who have X-linked disease is about:
  - 80%
  - 60%
  - 50%
  - 30%
  - 15%
3. The hearing loss of Alport syndrome
  - is congenital
  - is conductive
  - initially affects high frequency sounds
  - resolves following successful renal transplantation
  - is an uncommon feature of the disease
4. The prevalence of hematuria in females with X-linked Alport syndrome is
  - 25%
  - 40%
  - 60%
  - 75%
  - 95%
5. A small percentage of transplanted Alport patients develop
  - nephrotic syndrome
  - de novo membranous nephropathy
  - focal segmental glomerulosclerosis
  - anti-GBM nephritis
  - interstitial nephritis
